# Isolation of filamentous fungi from sputum in asthma is associated with reduced post-bronchodilator FEV_1_

**DOI:** 10.1111/j.1365-2222.2012.03987.x

**Published:** 2012-04-20

**Authors:** J Agbetile, A Fairs, D Desai, B Hargadon, M Bourne, K Mutalithas, R Edwards, J P Morley, W R Monteiro, N S Kulkarni, R H Green, I D Pavord, P Bradding, C E Brightling, A J Wardlaw, C H Pashley

**Affiliations:** 1 Department of Infection, Immunity and Inflammation, Institute for Lung Health, University of LeicesterLeicester, UK; 2 Department of Respiratory Medicine, Glenfield HospitalLeicester, UK

**Keywords:** airflow obstruction, asthma, fungal allergy, fungal culture

## Abstract

**Background:**

Fungal sensitization is common in severe asthma, but the clinical relevance of this and the relationship with airway colonization by fungi remain unclear. The range of fungi that may colonize the airways in asthma is unknown.

**Objective:**

To provide a comprehensive analysis on the range of filamentous fungi isolated in sputum from people with asthma and report the relationship with their clinico-immunological features of their disease.

**Methods:**

We recruited 126 subjects with a diagnosis of asthma, 94% with moderate-severe disease, and 18 healthy volunteers. At a single stable visit, subjects underwent spirometry; sputum fungal culture and a sputum cell differential count; skin prick testing to both common aeroallergens and an extended fungal panel; specific IgE to *Aspergillus fumigatus*. Fungi were identified by morphology and species identity was confirmed by sequencing. Four patients had allergic bronchopulmonary aspergillosis.

**Results:**

Forty-eight percent of asthma subjects were IgE-sensitized to one fungal allergen and 22% to ≥ 2. Twenty-seven different taxa of filamentous fungi were isolated from 54% of their sputa, more than one species being detected in 17%. This compared with 3 (17%) healthy controls culturing any fungus (*P* < 0.01). *Aspergillus* species were most frequently cultured in isolation followed by *Penicillium* species. Post-bronchodilator FEV_1_ (% predicted) in the subjects with asthma was 71(± 25) in those with a positive fungal culture vs. 83 (± 25) in those culture-negative, (*P* < 0.01).

**Conclusion and Clinical Relevance:**

Numerous thermotolerant fungi other than *A. fumigatus* can be cultured from sputum of people with moderate-to-severe asthma; a positive culture is associated with an impaired post-bronchodilator FEV_1_, which might be partly responsible for the development of fixed airflow obstruction in asthma. Sensitization to these fungi is also common.

## Introduction

Asthma is a condition characterized by variable airflow obstruction, airway hyperresponsiveness (AHR) and airway inflammation that is usually eosinophilic [1]. In many people with asthma, particularly in those with onset in childhood, there is a clear association with IgE sensitization to common aeroallergens (atopy) and it is assumed that the inflammatory process is due to IgE mediated mast cell activation and recruitment and activation of allergen-specific Th2 cells [2]. Fungal allergens including fungal fragments [3] are well recognized to induce florid hypersensitivity responses [4]. This can be due simply to exposure to high levels of fungal spores where, particularly in the case of *Alternaria*, they have been implicated as a cause of severe exacerbations and asthma deaths [5–7]. However, some people with airways disease appear susceptible to non-invasive colonization of the bronchial tree by thermotolerant filamentous fungi, in particular *Aspergillus fumigatus*. Colonization is associated with a syndrome usually seen in people with asthma and cystic fibrosis (CF), called allergic bronchopulmonary aspergillosis (ABPA). The criteria for the diagnosis of ABPA are fleeting shadows on the chest radiographs, a high total IgE, raised specific IgE or positive skin prick test (SPT) to *Aspergillus* allergens, raised specific IgG and bronchiectasis [8]. A peripheral blood eosinophilia and positive sputum culture are hallmarks of the syndrome, but are not essential for its diagnosis [9]. ABPA is most frequently associated with *A. fumigatus*; however, other *Aspergillus* species have been implicated including *A. niger*, *A. flavus*, *A. nidulans*, *A. oryzae* and *A. glaucus* [10]. In addition to *Aspergillus*, other fungal genera have been associated with clinical and radiological features similar to those of ABPA including *Penicillium, Candida*, *Curvularia*, *Drechslera, Fusarium*, *Geotrichum, Helminthosporium*, *Schizophyllum* and *Stemphylium* [10–12]. ABPA is regarded as being unusual; however, IgE sensitization to one or more fungal allergens is relatively common in severe asthma with up to 66% of people with severe asthma sensitized in a panel of six fungal allergens in one study [13]. The term severe asthma with fungal sensitization (SAFS) [4] has been coined to describe this phenotype, and the possibility that colonization with fungi might be pathogenic was suggested by the beneficial effect of a course of itraconazole [14]. In support of this concept, using a dedicated approach towards filamentous fungal detection, we were able to culture *A. fumigatus* from the sputum of 63% of asthmatics who were IgE-sensitized to the fungus on a single stable visit, in contrast to 31% of non-sensitized asthmatics and < 8% of healthy subjects. Patients sensitized to *A. fumigatus* had lower post-bronchodilator FEV_1_ than the non-sensitized group, suggesting that fungal colonization could contribute to the development of fixed airflow obstruction in asthma [15]. This idea is supported independently [16] and by data from people with cystic fibrosis (CF), suggesting a pathogenic role for *A. fumigatus* in the absence of a complete set of criteria to diagnose ABPA [17].

During the study investigating the relationship between *A. fumigatus* sensitization and lung function in asthma [15], we noticed that a significant number of other fungi were being cultured, particularly in those patients who were not sensitized to *A. fumigatus*. Unlike CF, [18, 19] there has been no comprehensive study looking at fungal colonization in moderate-to-severe asthmatics. Studies in asthma have focused mainly on patients suspected of having ABPA, and primarily reported only culture of *A. fumigatus*. The purpose of this study was therefore to fully characterize the fungal biota cultured from asthmatic sputum and examine the relationship between fungal culture and clinical features of asthma. We have shown that a positive sputum fungal culture and fungal sensitization independently lead to a trend in reduced lung function. The combination of fungal sensitization and sputum culture of any fungi is associated with a significant reduction in post-bronchodilator FEV_1_.

## Methods

### Study population

This was a cross-sectional study evaluating 126 subjects recruited over a 3-year period during 2008–2010 from the difficult asthma clinic in Glenfield General Hospital, Leicester, United Kingdom. The inclusion criteria included age ≥ 18 years, with a physician diagnosis of asthma (including 52 subjects at GINA step 5 and 67 subjects at step 4, Table 1). A diagnosis of asthma was based on clinical grounds by an experienced physician supported with either evidence of airflow obstruction on pre-bronchodilator FEV_1_, historical evidence of > 12% variability in their FEV_1_, a history of significant bronchodilator reversibility to 200 μg of inhaled albuterol after 15 min or evidence of hyperresponsiveness on methacholine challenge with PC20 < 8 mg/mL. Eighteen healthy volunteers served as controls and included members of the public and staff at Glenfield hospital. ABPA was diagnosed using widely accepted criteria [8, 9, 20].

**Table 1:** Demographic data

	Asthma patients (*n* = 126)				Comparing three groups
			
	No fungi cultured (*n* = 58)	Any fungi (*n* = 68)	*P-*value	Healthy controls (*n* = 18)*	*P-*value
Age in years (range)	55 (21–84)	58 (24–83)	0.23	40 (21–67)	< 0.001
Smoking history (pack years)†	0 (0–4)	0 (0–10)	0.51	0 (0–3)	0.44
Gender (male)	41%	53%	0.20	50%	0.42
Serum total IgE kU/L†	159 (43–494)	207 (89–718)	0.08	31 (9–50)	< 0.001
Atopic§	55%	61%	0.57	17%	0.01
Age of asthma onset, years†	34 (9.5–47.25)	25 (5.25–46)	0.65	-	-
Duration of asthma, years†	22 (10.75–42.5)	23 (7–41.75)	0.89	-	-
FEV_1_% of predicted, post-bronchodilator	82.8 (24.8)‖	70.8 (25.4)‖	< 0.01	111.6 (11.0)‖	< 0.001
Volume change post-bronchodilator, (mL)†	100 (50–250)	50 (0–150)	0.01	-	-
Fungal sensitization, (any)	38%	56%	0.08	6%	< 0.01
*• Aspergillus fumigatus* (positive/n)	17/58	35/68	0.02	0/18	
*• Penicillium chrysogenum*	5/36	17/48	0.04	0/12	
*• Botrytis cinerea*	3/31	8/41	0.30	0/12	
*• Alternaria* alternata	6/39	11/58	0.80	1/8	
*• Cladosporium herbarum*	7/38	13/57	0.80	0/8	
GINA treatment					
GINA 5	38%	44%	0.58	-	-
GINA 4	55%	51%			
Inhaled corticosteroid dose (μg)†¶	1600 (800–2000)	2000 (1600–2000)	0.04	-	-
Number with bronchiectasis, n (%)	17 (35)	32 (51)	0.06	-	-
Total cell count × 10^3^ mg of sputum‡	3.151	3.451	0.91	-	-
Sputum neutrophil (%)‡ (95% CI)	58.09 (48.8–69.2)	51.65 (42.5–62.8)	0.47	-	-
Sputum eosinophil (%)‡(95% CI)	2.52 (1.5–4.2)	2.09 (1.4–3.2)	0.61	-	-

* Three subjects had positive fungal cultures.

† median (IQR),

‡ geometric mean

§ Assessed by SPT ≥ 3 mm or specific IgE to common aeroallergens.

¶ Beclometasone Diproprionate equivalent.

‖ Post-test comparison *P* < 0.05.

The incidence of *A. fumigatus* culture for 79 of the asthmatics and 14 of the healthy volunteers was reported previously [15].

The study was approved by the Leicestershire and Rutland ethics committee and all subjects gave their written, informed consent.

### Clinical assessment

Subjects were seen at a single stable visit and clinical data collected including gender, smoking history, age of asthma onset, physiological parameters of spirometry, sputum for differential cell counts, and fungal culture, prescribed inhaled and systemic corticosteroid therapy. Inhaled corticosteroid (ICS) doses were standardized to Beclometasone diproprionate (BDP)**-**HFA equivalent [21].

### Spirometry

All patients underwent standard spirometry. Post-bronchodilator measurements were recorded 15 min after 200 μg of inhaled albuterol according to ATS/ERS guidelines [22]. FEV_1_ was calculated according to the best successive readings in 100 mL using a dry bellows spirometer (Vitalograph Ltd, Maids Moreton, UK) [23, 24]. Sputum was obtained either spontaneously or by induction using hypertonic saline [25, 26].

### Fungal culture and identification

Selected sputum plugs were plated directly onto fungal-specific culture media as previously described [15] and incubated at 37°C for up to 7 days. Fungi were identified based on macroscopic and microscopic morphology [27]. Species identity was confirmed by sequencing either the large subunit [28] or the internal transcribed spacer region 1 [29] of the nuclear ribosomal operon, using PCR conditions as previously described [28]. Total genomic DNA was extracted from pure subcultures using the DNeasy plant mini kit (Qiagen, West Sussex, UK) following manufacturer's instructions, with the inclusion of a bead-beating step (BioSpec mini bead beater, Bartlesville, OK, USA). Sequences were determined using BigDye-Terminator v3.1 chemistry with 3730 sequencers (Applied Biosystems, Warrington, UK). Sequence data were manually inspected and trimmed, with closest taxonomic match determined by comparison with known sequences in GenBank (March 2010) using the BLAST_N_ database search method.

### Allergy testing

Sensitization was evaluated using SPT (Alk-Abello, Hørsholm, Denmark) to common aeroallergens including cat, dog, *Dermatophagoides pteronyssinus* and grass pollen with a fungal panel including *A. fumigatus*, *Alternaria alternata*, *Botrytis cinerea, Cladosporium herbarum* and *Penicillium chrysogenum*. A positive test was defined as a weal of ≥ 3 mm above negative control. Total IgE and specific IgE to *A. fumigatus* were quantified using the Unicap250 system (Pharmacia, Milton Keynes, UK) with specific IgE against *A. fumigatus* > 0.35 kU/L classed as positive.

### Bronchiectasis

Cross-sectional imaging was performed for clinical reasons in 116 subjects. Computed tomography thoracic scans of the thorax were acquired with a Picker PQS (Picker International, Cleveland, OH, USA) or Siemens sensation 16 scanner (Siemens Healthcare, Knoxville, TN, USA). A diagnosis of bronchiectasis was based on the radiologist's report [30].

### Macrophage culture and influence of glucocorticoids

*A. fumigatus* NCPF (National Collection of Pathogenic Fungi, UK) 7097 conidia were harvested in HBSS and adjusted to stock concentrations of 1 × 10^8^ conidia/mL. Bronchoalveolar macrophages were obtained from three lung carcinoma patients undergoing upper lung lobectomies (two female, one male; mean age 63). Lung filtrate was washed twice with Hank's balanced salt solution (HBSS; Sigma, Gillingham, UK), red blood cells lysed and cells resuspended in dulbecco's modified eagle's medium (DMEM; Invitrogen, Paisley, UK) supplemented with 10% fetal bovine serum (Invitrogen), 1% non-essential amino acids (Fisher Scientific UK Ltd, Loughborough, UK), penicillin (50 U/mL) and streptomycin (50 μg/mL). Viability and purity of macrophages were assessed by the trypan blue exclusion method and kimura stain respectively. 4 × 10^5^ macrophages were seeded onto sterile glass coverslips in 24-well plates and incubated overnight (37°C, 5% C0_2_). Adherent macrophages were washed twice with HBSS and incubated in media containing varying concentrations of dexamethasone (10^−6^–10^−9^ m diluted in HBSS) in triplicate wells per dilution, with HBSS as negative control. Following 24 h incubation, (shown previously to enable suppression of macrophage activity [31]), macrophages were challenged with 4 × 10^6^ *A. fumigatus* conidia and incubated for 45 min (37°C, 5% C0_2_)_._ Extracellular conidia were removed by washing and macrophages incubated for 4 h. Coverslips were then washed, fixed in methanol and stained with Rapi–Diff (Biostain Ready Reagents Ltd, Manchester, UK). The phagocytic index was assessed by microscopic analysis of 200 macrophages per coverslip in triplicate for each treatment and non-treatment control, with lab personnel blinded to treatment conditions.

### Statistical analysis

Data were analysed using GraphPad (Version 5; GraphPad Software Inc, La Jolla, CA, USA) and SPSS for Windows (Version 11.0; SPSS, Inc., Chicago, IL, USA). Parametric data were expressed as means ± standard deviation (SD) and analysed by unpaired student-*t*-test. Between-group comparisons were analysed by Bonferonni-corrected anova. Macrophage data were analysed by repeated-measures anova (Bonferonni-corrected). Non-parametric data were expressed as medians with interquartile ranges (IQR) and analysed using Mann–Whitney, chi-squared, Fisher's exact and Dunn-corrected Kruskal–Wallis tests.

## Results

### Demographic characteristics

The demographic characteristics of the subjects with asthma and the healthy controls are shown in Table 1. The mean (± SD) age of subjects with asthma was 56 ± 13.4 years, with median (IQR) age of onset 33 (7–46.3) years. The asthma cohort was matched with the healthy volunteers with respect to smoking history and gender. Asthma subjects were, however, significantly older with higher rates of atopy than controls. Forty-eight percent of asthma subjects had evidence of IgE sensitization to commercial extracts of at least one of a panel of five fungi, 22% to two or more fungi.

Ninty-four percent of asthma subjects required Global Initiative for Asthma (GINA 4) or greater treatment and within the population, there was a significant degree of fixed airflow obstruction as evidenced by impaired post-bronchodilator FEV_1_. Forty-two percent of subjects with asthma scanned had bronchiectasis, although only four patients met criteria diagnostic of allergic bronchopulmonary aspergillosis.

### Fungal culture and sensitization

A significantly higher rate of fungal culture was detected in the sputum of subjects with asthma (54%) compared with healthy controls (17%, *P* < 0.01). Within the group of culture-positive asthmatics, *A. fumigatus* was the sole fungus isolated from 54% (37/68) of subjects; over a quarter (27%) cultured both *A. fumigatus* and at least one other fungus; and nearly a fifth (19%) cultured at least one fungus without co-culture of *A. fumigatus*.

A total of 97 fungal cultures representing 27 different taxa of filamentous fungi were obtained from 68 asthmatic sputa. The majority (92%) were from the genera *Aspergillus* and *Penicillium*, with *A. fumigatus* representing 57% of isolates (Table 2). In addition to *A. fumigatus*, a further 18 different *Aspergillus* or *Penicillium* species or groups of closely related species were detected, eight of which represented nine isolates where *A. fumigatus* was not co-cultured.

**Table 2:** Identification and incidence of filamentous fungi cultured from the sputum of asthmatics and healthy controls. Isolates were either the only filamentous fungi cultured (mono), grew in co-culture with *A. fumigatus* (co-Af), or in co-culture with other filamentous fungi listed but no *A. fumigatus* (co-other)

			Asthmatic (*n* = 126)			Healthy control (*n* = 18)		
				
Class	Genus	Species/taxonomic identifier	Mono	Co-Af	Co-other	Mono	Co-Af	Co-other
Eurotiomycetes	*Aspergillus*	*A. fumigatus*	37		18	1		
		*A. fischeri* var. *glaber*		1				
		*A. niger* complex	2	4	1			
		*A. terreus*		1				
		*A. ustus*			1			
		Section Flavi (species undetermined)		2				
		Section Nidulantes (species undetermined)	1	1				
		Species undetermined		1				
	*Penicillium*	*P. brasilianum*	1					1
		*P. capsulatum,*			1			
		*P. chrysogenum* or *P. gladioli*	1					
		*P. citrinum*	1					
		*P. citrinum* or *P. griseofulvum*			1			
		*P. diversum*		1				
		*P. verruculosum*	1					
		*P. marneffei*	1					
		*P. simplicissimum or brasilianum*						1
		*P. piceum*	1	3	3			
		*P. pinophilum*		1				
		Subgenus *Penicillium* (species undetermined)		2				
		Species undetermined		1				
	*Paecilomyces*	*P. variotii*		1				
		*Thermoascus crustaceus* (*Paecilomyces* teleomorph)		2				
	*Gymnascella*	*G. citrina*			1			
Zygomycota (class undetermined)	*Rhizomucor*	*R. miehei*		1				
	Genus undetermined	Species undetermined		1				
Agaricomycetes	*Coprinus*	*C. xanthothrix*						1
	Genus undetermined	Species undetermined		1				
Sordariomycetes	*Chaetomium*	*C. bostrychodes*				1		
	Genus undetermined	Species undetermined	1					
No filamentous fungal growth			58			15		

The rate of sensitization to *Aspergillus* and *Penicillium* species combined was significantly higher in those with a positive fungal culture than those without (54% vs. 31%, *P* = 0.01); this association was not seen for *Alternaria alternata, Cladosporium herbarum and Botrytis cinerea,* which were not cultured.

### Clinical correlations

**Fig. 1:**
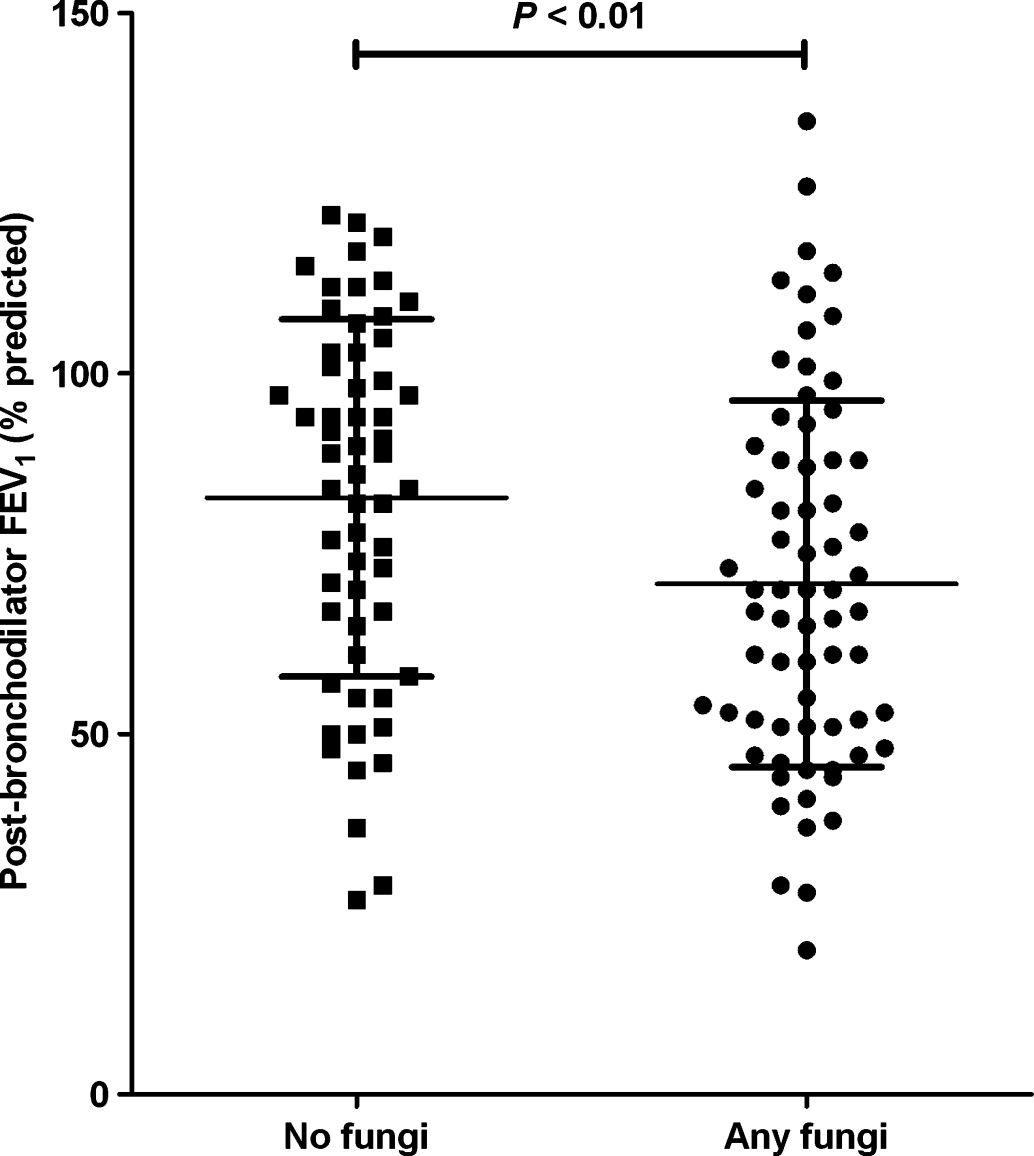
A comparison of the mean (SD) percent predicted post-bronchodilator FEV_1_ in subjects with asthma and a negative fungal sputum culture vs. those with a positive culture. Culture-positive asthmatics had a significantly lower level of lung function (70.8% ± 25.4 vs. 82.8% ± 24.8: *P* < 0.01) than culture-negative asthmatics.

**Fig. 2:**
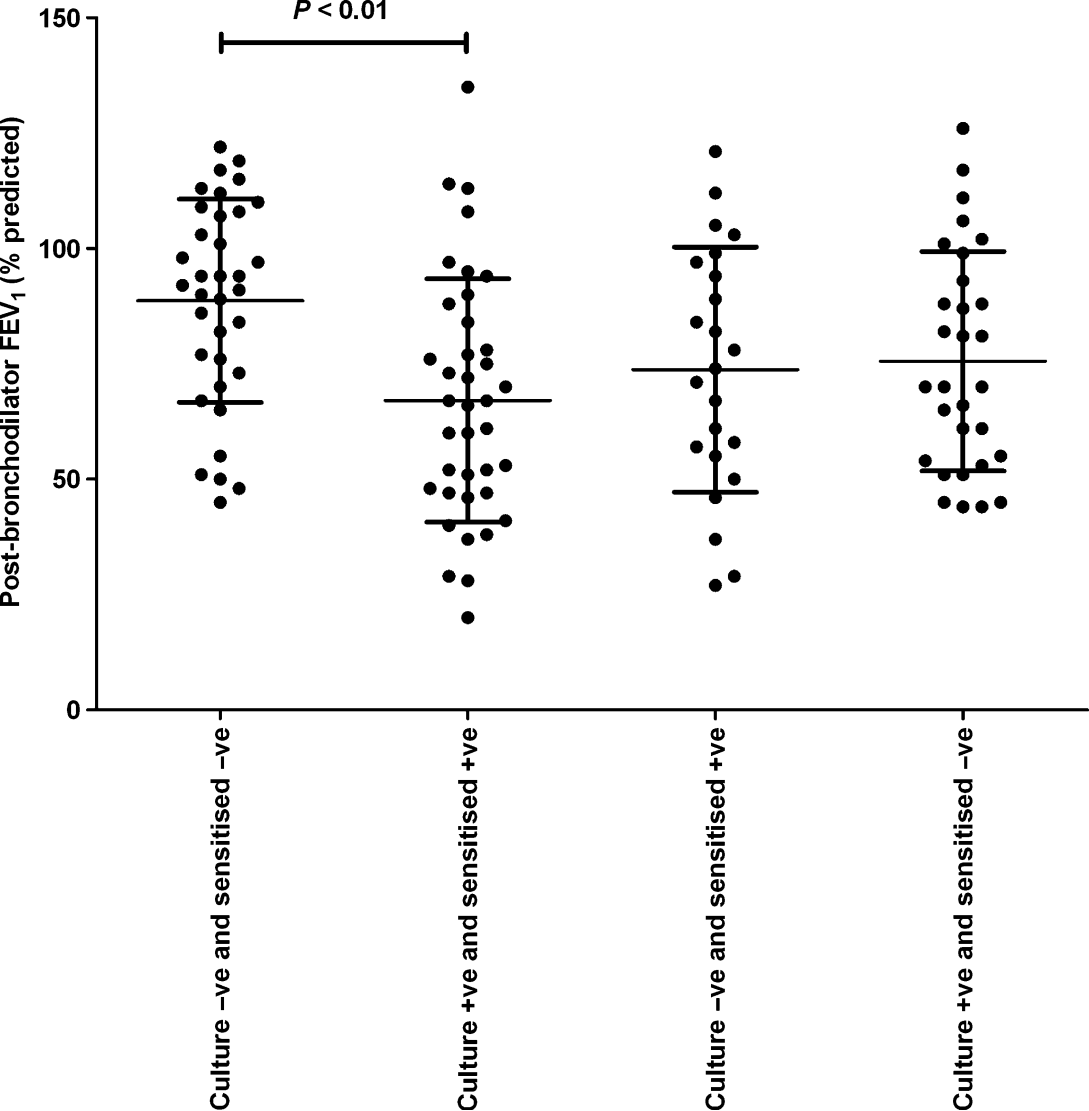
A comparison of the mean (SD) percent predicted post-bronchodilator FEV_1_ between subjects with asthma who were sputum fungal culture-negative (C−) and were not sensitized (S−) to any fungi from a panel of five tested, culture-positive (C+) and sensitized (S+) to any mould, culture-negative and not sensitized and culture-positive and not sensitized. The non-sensitized culture-negative group had significantly better lung function than the sensitized and culture-positive group. (88.7% ± 22.0 vs. 67.1% ± 26.4); *P* < 0.01).

There was a lower post-bronchodilator FEV_1_ in subjects with a positive sputum fungal culture compared with those without (70.8% ± 25.4 vs. 82.8% ± 24.8: *P* < 0.01: Table 1 and Fig. 1). Consistent with there being a greater degree of fixed airflow obstruction in the fungal culture-positive group, there was significantly less reversibility to short-acting bronchodilators (50 mL vs. 100 mL; *P* = 0.01) despite being on higher doses of inhaled steroids. There was also a trend towards more subjects having bronchiectasis in the fungal culture-positive group, but this did not quite reach significance. There was no significant difference in the age of onset, duration of asthma, atopic status (not taking into account fungal sensitization), or sputum differential count between the two groups. We have previously noted that sensitization to *A. fumigatus* was associated with impaired lung function. We therefore investigated the combined effect of fungal sensitization and culture. Subjects without sensitization or culture had significantly better % predicted post-bronchodilator FEV_1_ compared with subjects who were both sensitized and culture-positive (88.7 ± 22 vs. 67.1 ± 26.4: *P* < 0.01, Fig. 2). This was a more marked difference than culture alone. Subjects who had either sensitization or a positive culture had an intermediate predicted FEV_1_%, which was not significantly different from the double negative group (culture-positive, sensitization-negative 75.6 ± 23.8: culture-negative, sensitization-positive 73.7 ± 26.6).

### Effect of corticosteroids on the phagocytic index of macrophages

**Fig. 3:**
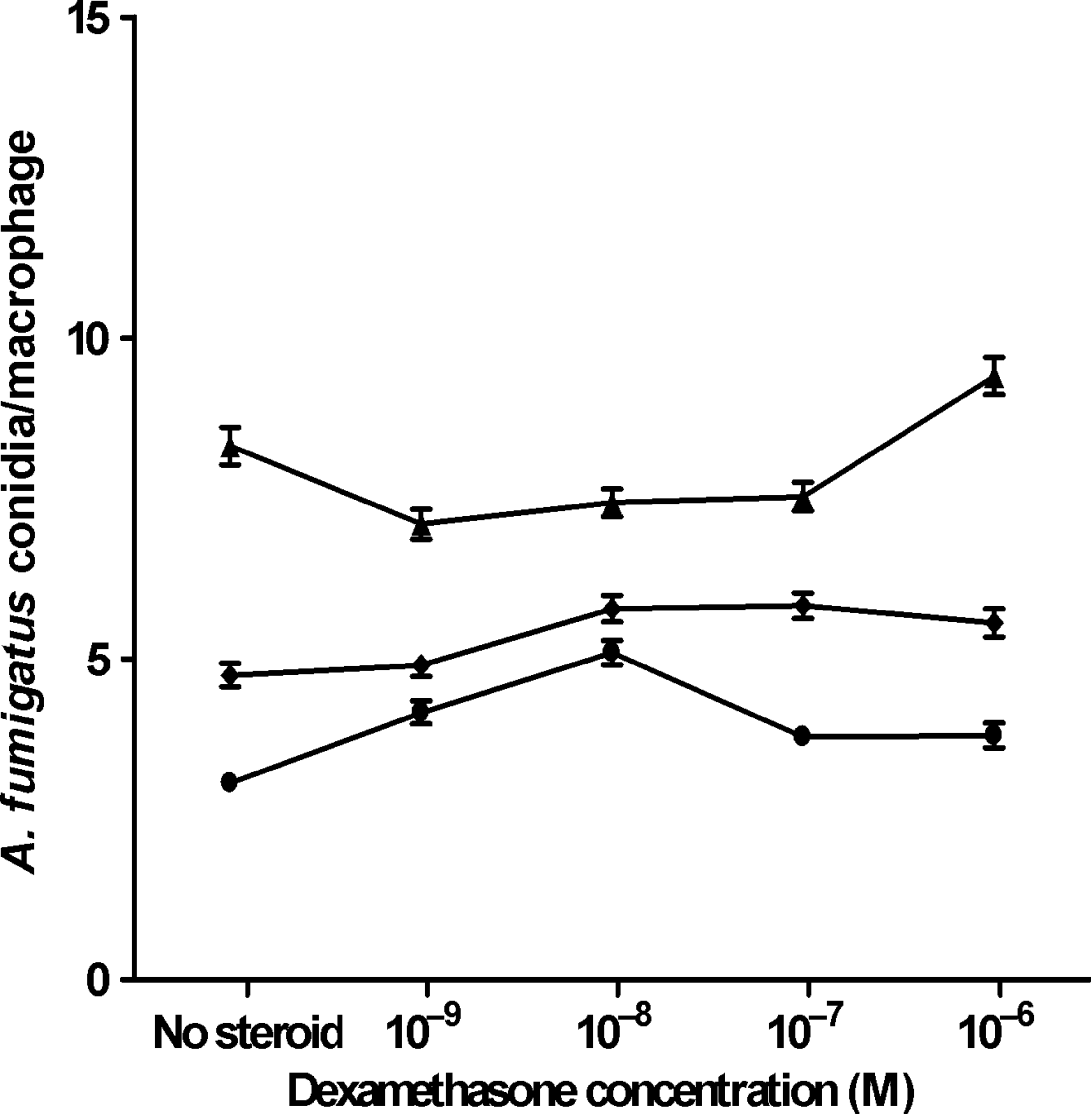
The phagocytic index of macrophages targeting *A. fumigatus*, dependent on concentration of dexamethasone, showing the mean and standard error from three separate experiments. Individual plots represent analysis of 200 macrophages from triplicate wells per experiment. Different symbols denote the three patients.

Concentrations of dexamethasone within the therapeutic range (10^−6^–10^−9^ M) did not affect the phagocytic index of bronchoalveolar macrophages engulfing *A. fumigatus* conidia *in vitro* (Fig. 3). There was no difference in the viability of steroid-treated or -untreated macrophages, in the presence or absence of *A. fumigatus* conidia, with mean macrophage viability of 94%.

## Discussion

This is the first study that we are aware of that has systematically detailed the fungal biota in sputum from a large number of subjects with asthma. We have made three striking and novel observations: First, that a positive sputum culture for a variety of filamentous fungi, other than *Aspergillus fumigatus,* is very common in moderate-to-severe asthma with over half the subjects having a positive sputum culture for one or more fungi on a single stable visit; secondly that most of the cultured fungi are from the allergenic fungal genera *Aspergillus* and *Penicillium* with *A. fumigatus* being the single most common species; and thirdly, that a positive sputum fungal culture is associated with impaired bronchodilator FEV_1_, supporting the hypothesis that fungal colonization of the airways causes the development of fixed airflow obstruction in asthma. The study unfortunately is not powered enough to show a significant difference in lung function between the various types of fungi. In our experience, it is unusual in asthma for a positive sputum culture for fungi to be reported in a routinely processed sample, even in subjects who fulfil all the criteria for ABPA. On those occasions when there is a positive report, it is invariably *A. fumigatus*. Part of the reason for this is that sputum is not routinely analysed for fungi in asthma where adequate spontaneous samples are infrequently produced. Furthermore, in those infrequent cases where microbiological analysis is sought, samples are usually only sent when there is sputum purulence and bacterial infection is being considered. Fungal culture is mainly considered when ABPA is suspected, although culture of fungi is of no major diagnostic criteria in any of the most widely used studies [8, 9, 20]. We propose, on the basis of our data, that a full appreciation of the potential role of filamentous fungi in asthma requires a focused approach to fungal culture. In support of this, we have recently undertaken a study comparing the way in which sputum is processed in our routine national health service (NHS) clinical microbiology laboratory, which follows a national standard method 32 compared with our research-based technique [15]. We found that the routine method is insensitive, even when a fungal culture is specifically requested, mainly because the sample is much diluted compared with our approach [33].

The high rate of culture positivity in our subjects raises the question of the specificity of our technique. However, only three of eighteen healthy controls (17%) cultured fungi in their sputum. Sputum samples from healthy controls and asthma subjects were treated identically and laboratory staff were blinded to the subjects' medical status, ruling out environmental contamination as the cause of the difference in culture rates observed. Although a positive sputum culture may indicate colonization of the airways suggesting that the fungus is growing non-invasively in the bronchial lining fluid, it could be obtained from the upper airway or the result of germination of an inhaled spore [34]. Removal of saliva and selection of sputum plugs, combined with a marked reduction in positive culture rates from healthy subjects, suggest that it is unlikely that the fungi cultured are coming from the upper airway.

There was a difference in age between the healthy controls and asthmatics and therefore we cannot exclude the possibility that rates of culture increase with lung age, although we feel that it is unlikely to explain the difference seen. Although we found high rates of positive culture, it is possible that molecular techniques such as the polymerase chain reaction (PCR) would result in even higher positive rates of detection, and this approach needs to be compared with our culture method.

The majority of patients recruited into the study were on high-dose inhaled steroids, and asthma patients with a positive sputum culture for filamentous fungi were prescribed significantly higher doses of inhaled steroids. Glucocorticoids have been reported to elicit immunosuppressive effects including impaired activity of lymphocytes, neutrophils and macrophages, leading to increased susceptibility to fungal infection [35]. Treatment with corticosteroids has been shown in bronchoalveolar macrophages from both mice [36] and humans [31] to cause impaired clearance and killing of *Aspergillus fumigatus* conidia, and has also been associated with enhanced growth of *Aspergillus* species [37]. We found no significant effect of therapeutic levels of glucocorticoids on the phagocytic index of macrophages, consistent with earlier studies [31]; however, it has been reported that whilst the phagocytic index of macrophages remains unchanged, the ability of macrophages to actually kill spores is impeded by glucocorticoid treatments [31]. Future studies investigating the prescribed dose and metabolism of corticosteroids, in relation to clearance of inhaled conidia from the lung and rates of fungal culture from sputum in asthma, will be required to fully understand the role of steroids in rates of airways colonization.

The data we obtained were on a single visit and we do not have data on reproducibility; however, we have previously shown repeatability of *A. fumigatus* culture to be reasonably good [15]. A caveat of our culture approach is that we cannot readily quantify the amount of fungi in a sputum sample and quantitative PCR (QPCR) may be an advantage in this respect; however, QPCR is only able to detect the fungus to which specific primers have been designed.

Most of the fungi cultured were from the *Aspergillus* and *Penicillium* genera. Many mechanisms enable these fungi to colonize the human airway. Small spore size permits them to bypass the filtering system of the upper airways and continue deposition in the distal small airways. In addition, many members have thermotolerant growth properties allowing them to grow at body temperature in the airways.

In the context of ABPA and CF, other species of *Aspergillus* (most commonly *A. niger* and *A. flavus)* have been reported in a minority of patients. *Penicillium* spp. have been reported in as many as 9% of CF patients [38], but are not routinely distinguished to species and are often regarded as a contaminant [39]. In our study, 13% of subjects with asthma cultured one or more species of *Penicillium*, 7% in the absence of co-culture with *A. fumigatus*. *P. piceum* was the most common species of *Penicillium* cultured from people with asthma in our study, and is a member of the *P. marneffei* complex [40], an emerging opportunistic human pathogen [41]. Fungi from the genera *Paecilomyces, Rhizomucor, Coprinus* and *Chaetomium,* from which we isolated species in this study, have been described in case reports of pathogenic infection [42], mucormycosis [43], pulmonary infection [44] and invasive mycotic infections [45] respectively. This suggests that isolates from these fungal genera should not necessarily be disregarded as being clinically significant.

*Aspergillus* and *Penicillium* are also the most common indoor fungi with spores present in significant numbers even in houses without obvious damp [3, 46, 47]. Whether people who culture fungi from their sputum are exposed to higher numbers of spores in the home remains unclear. A small pilot study of thirteen homes of people with and without ABPA concluded that host susceptibility was more important than environmental exposure [48]; however, further studies would be required to confirm this.

A number of subjects had more than one fungus isolated from their sputum suggesting either heavy exposure or, perhaps more likely, a defect in host defence against fungi, making them susceptible to colonization. Such defence involves a combination of innate and adaptive immunity [49], and the extent to which there is deficiency in any of these pathways in some people with asthma is unknown. The development of IgE sensitization in those not currently sensitized but colonized is also worthy of further study.

Allergic bronchopulmonary mycoses are associated with IgE sensitization. Unfortunately, we were not able to obtain a complete data set for fungal sensitization, in part due to a lack of immunological testing solutions. However, with this caveat, those with a positive culture were significantly more likely to be sensitized to *A. fumigatus* or *P. chrysogenum* (the only species within the *Aspergillus and Penicillium* genera with commercially available skin test reagents). No such relationship between sensitization and culture was seen for the non-thermophilic *Alternaria, Cladosporium* or *Botrytis,* which act as aeroallergens, but do not colonize the airways, supporting the idea that colonization and sensitization are directly related. We have previously reported that sensitization to *A. fumigatus* alone is associated with an increased rate of positive sputum culture for *A. fumigatus* [15]. Linking sensitization and culture to the panel of fungi cultured in this study is more problematic due to the lack of reagents for the majority of species identified. Furthermore, the degree of cross-sensitization between species is not clear [50]. It is possible if we were able to specifically test for IgE sensitization to the full panel of fungi, then the rates of sensitization in the positive sputum group would be even higher.

The major question arising from our study is the extent to which fungal colonization is clinically important. Are the fungi commensals commonly found in damaged lungs or do they have a pathogenic role? Very few of the subjects, four, in our study fulfilled all the criteria for classical ABPA, where there is good evidence for a tissue-damaging role of chronic fungal colonization with *A. fumigatus*. In support of the idea that the fungi are pathogenic, we have found an association between a positive fungal culture and reduced post-bronchodilator FEV_1_. This extends the observation of our previous study where IgE sensitization to *A. fumigatus* was associated with impaired lung function, but where culture for *A. fumigatus* was only weakly associated as part of a multi-variable analysis [15]. The larger numbers of subjects in this study and the extension to cover any filamentous fungus have resulted in a negative association between lung function and culture, irrespective of sensitization status. If a subject is also IgE-sensitized to fungal allergens, then the effect on lung function appears greater than for culture alone (12% vs. 22%), suggesting that both factors are involved in impairment of lung function. We do not have enough subjects to explore the effects of a filamentous species other than *A. fumigatus* cultured in isolation with lung function, although there was a trend to lower lung function in this group compared with the no-culture group (data not shown). A longitudinal study of patients representing the full spectrum of asthma, with and without fungal sensitization, is the only means of fully addressing the question as to whether the relationship between fungal colonization and reduced lung function is causal; or that saprophytic fungal colonization is just more likely in already more severely damaged lungs.

We previously noted a relationship between sputum neutrophilia and lung function [51], but this relationship was not apparent in this study. Fungi produce a range of toxins, which are potentially tissue-damaging. In addition, where sensitization is present, they can promote an inflammatory response through allergic mechanisms as well as potentially by an autoimmune-like process caused by cross-reaction between fungal and human antigens [52, 53]. It is therefore very plausible that chronic colonization of the airways with a range of fungi could promote IgE sensitization and in turn lead to chronic airway damage. Our work would suggest that identification of airway damage in asthma and the presence of fungi in the airway using optimal techniques is an important priority in asthma management.

In summary, we have found high rates of fungal culture with sensitization to mainly the *Aspergillus* and *Penicillium* genera from subjects with moderate-to-severe asthma who generally do not fulfil the criteria for ABPA. A positive sputum culture is associated with fixed airflow obstruction, supporting the hypothesis that this asthma phenotype is caused by chronic fungal airway colonization.
